# Elucidation of the ‘Honeycrisp’ pedigree through haplotype analysis with a multi-family integrated SNP linkage map and a large apple (*Malus*×*domestica*) pedigree-connected SNP data set

**DOI:** 10.1038/hortres.2017.3

**Published:** 2017-02-22

**Authors:** Nicholas P Howard, Eric van de Weg, David S Bedford, Cameron P Peace, Stijn Vanderzande, Matthew D Clark, Soon Li Teh, Lichun Cai, James J Luby

**Affiliations:** 1 Department of Horticultural Science, University of Minnesota, St Paul, MN 55104, USA; 2 Department of Plant Breeding, Wageningen University and Research, Wageningen 6700AJ, The Netherlands; 3 Department of Horticulture and Landscape Architecture, Washington State University, Pullman, WA 99164, USA; 4 Department of Horticulture, Michigan State University, East Lansing, MI 48824, USA

## Abstract

The apple (*Malus×domestica*) cultivar Honeycrisp has become important economically and as a breeding parent. An earlier study with SSR markers indicated the original recorded pedigree of ‘Honeycrisp’ was incorrect and ‘Keepsake’ was identified as one putative parent, the other being unknown. The objective of this study was to verify ‘Keepsake’ as a parent and identify and genetically describe the unknown parent and its grandparents. A multi-family based dense and high-quality integrated SNP map was created using the apple 8 K Illumina Infinium SNP array. This map was used alongside a large pedigree-connected data set from the RosBREED project to build extended SNP haplotypes and to identify pedigree relationships. ‘Keepsake’ was verified as one parent of ‘Honeycrisp’ and ‘Duchess of Oldenburg’ and ‘Golden Delicious’ were identified as grandparents through the unknown parent. Following this finding, siblings of ‘Honeycrisp’ were identified using the SNP data. Breeding records from several of these siblings suggested that the previously unreported parent is a University of Minnesota selection, MN1627. This selection is no longer available, but now is genetically described through imputed SNP haplotypes. We also present the mosaic grandparental composition of ‘Honeycrisp’ for each of its 17 chromosome pairs. This new pedigree and genetic information will be useful in future pedigree-based genetic studies to connect ‘Honeycrisp’ with other cultivars used widely in apple breeding programs. The created SNP linkage map will benefit future research using the data from the Illumina apple 8 and 20 K and Affymetrix 480 K SNP arrays.

## INTRODUCTION

‘Honeycrisp’ has emerged as an economically lucrative apple (*Malus*×*domesti* *ca*) cultivar in North America that has steadily gained market share^1^ since its introduction by the University of Minnesota (UMN) apple breeding program in 1991.^2^ It has also been an increasingly important parent due to its reportedly ultracrisp texture,^3^ its ability to retain this high level of crispness in storage^4–6^ and its resistance to apple scab.^7^ ‘Honeycrisp’ is a parent of multiple commercially released cultivars including ‘Minneiska’,^8^ ‘New York 1’,^9^ ‘CN B60’,^10^ ‘CN 121’,^11^ ‘DS 22’,^12^ ‘WA 38’,^13^ ‘MAIA1’,^14^ ‘MN55’^15^ and new Chinese cultivars, with many additional offspring under testing around the world as advanced breeding selections. The importance of ‘Honeycrisp’ in production and breeding has led to many studies of the physiology and genetics of the variety. It was quickly discovered that the originally recorded parentage, ‘Honeygold’×‘Macoun’,^2^ did not complement phenotype information for several key traits.^4^ As DNA markers became increasingly available for apple, analysis of simple sequence repeat (SSR) markers confirmed that the recorded parentage was incorrect.^16^ Breeding records of the UMN apple breeding program indicated that the cross that begat ‘Honeycrisp’ was likely made around 1960, but a lack of records from this period rendered direct validation of parentage inconclusive.

‘Honeycrisp’ was first selected from a seedling block as MN1711 in 1974. The original tree had been discarded due to winter injury in 1977. Four clonal propagules that were planted in a testing orchard had also been flagged for removal, but they were spared and kept for further evaluation by the newly appointed breeder, David Bedford, because the trees had been planted in a poor site and there had been insufficient evaluation notes taken for the selection. No additional records were available that could be used to identify likely parents and it had been speculated that the original parent might have been an older selection from the program that was discarded.^16^ ‘Keepsake’ was identified as one of the parents^16^ based on results from 11 SSR markers, but the identity of the second parent has remained a mystery.

Past studies evaluating pedigree relationships in apple have been conducted with a range of genetic marker types including SNPs in chloroplast DNA,^17^ randomly amplified polymorphic DNA^18^ and SSRs.^16,19^ Recently, single-nucleotide polymorphism (SNP) arrays have become available for apple,^20–22^ making possible the generation of a large amount of standardized genetic data that are useful for relationship analyses. SNP array-based parental testing has been successfully used to identify ‘Common Antonovka’ as the parent of a series of selections that had been widely used in the breeding for scab resistance.^23^ In sweet cherry (*Prunus avium*), SNP array data have been successfully used for the identification of the previously unknown paternal parent of the important U.S. cultivar Bing.^24^ The SNP arrays and wealth of genetic data generated through the RosBREED project, funded in part by the USDA-Specialty Crop Research Initiative,^25^ has provided an excellent opportunity for this type of relationship testing.

The objective of this study was to test an earlier hypothesis that ‘Keepsake’ is one parent of ‘Honeycrisp’^16^ and to identify and genetically describe the unknown parent and grandparents of ‘Honeycrisp’ using a much larger set of DNA markers than was previously available with this germplasm. This work relied on the adequate phasing of thousands of SNP markers, which was made possible through the development and use of a high-quality integrated SNP linkage map, also described in this study, that was developed by closely following many methods used by Di Pierro *et al.* ^26^

## MATERIALS AND METHODS

### Genetic map creation

Five families with ‘Honeycrisp’ as a common parent were used in the creation of the integrated genetic map used in this study. These families were described by McKay *et al.* ^27^ and planted as clonal replicates on ‘Budagovsky 9’ rootstocks at the UMN’s Horticultural Research Center in Chanhassen, Minnesota, USA in 2010. These families were ‘Honeycrisp’×MN1764 (*n*=156), ‘Honeycrisp’×‘Monark’ (*n*=91), ‘Honeycrisp’×‘Pitmaston Pineapple’ (*n*=60), ‘Honeycrisp’×‘Jonafree’ (*n*=57) and ‘Honeycrisp’×MN1702 (*n*=49). Leaf samples were collected from these individuals for extraction of DNA that was hybridized onto the International RosBREED SNP Consortium 8 K Illumina Infinium array v1^20^ following the methods outlined in Clark *et al*.^28^ The first two of these five populations were previously used in the creation of an earlier ‘Honeycrisp’ consensus genetic map.^28^ The raw iScan data output from the apple 8 K SNP array from all individuals was imported into the genotyping module of GenomeStudio software version 1.9.4 for SNP scoring (Illumina, Inc., San Diego, CA, USA). SNP scores were coded as A and B in GenomeStudio. SNP probe sequences and targeted polymorphic nucleotides for the A/B scoring for each SNP in the 8 K SNP array can be found in the Supplementary Material of Chagné *et al*.^20^ and at the Genome Database for Rosaceae,^29^ as well as in our Supplementary Table 1 (Flanking_Sequence column). SNP scoring was conducted following Clark *et al*.^28^ with the exception that all SNPs were visually inspected and manually clustered when necessary regardless of GenTrain scores and heterozygote frequencies. SNP scores from each individual in each population were compared to both its parents’ SNP scores to ensure that the listed parentage was correct. Seedling individuals that had >200 inheritance errors out of the total 8788 SNPs (2.3% error rate) on the apple 8 K SNP array were removed from analysis. These types of inheritance errors, referred to as Mendelian-inconsistent errors (also known as Mendelian errors),^30^ were recorded as instances where an individual had a SNP score that could not have arisen given the SNP scores of its parents or offspring. For example, if an individual’s SNP score at an individual locus was *AA* and one or both of its parents’ SNP scores were *BB* this would count as an inheritance error as either the individual and/or its parent(s) must have incorrect SNP scores. Another type of observed Mendelian-inconsistent error was where an individual’s SNP score was *AB* and its parents’ SNP scores were both either *AA* or *BB*.

SNPs with >15% missing data and SNPs that had apparent null alleles in one or more parents were excluded from analysis. Marker segregation classes were then generated with the remaining SNPs by evaluating segregation ratios within each family. Genotypic score classes that comprised at least 2% of the individuals within a family were taken into account when defining the segregation classifications in order to account for possible segregation distortion. Individual SNP scores that did not follow the overall Mendelian segregation classifications for <2% of the individuals within the family were recoded as missing data. After applying these rules, the marker segregation classes of seedlings from each family were compared to parental SNP scores. Parental SNP scores that still did not match the segregation classes of seedlings were altered to fit the segregation classes if a change in ‘Honeycrisp’ resulted in agreement in segregation classification across all five populations consistent with the SNP score of the other parent in each family or if a change in the other parent could resolve the inconsistency between it and the segregation of its progeny. These steps were conducted in this order because the original GenomeStudio project was not available for re-examination of the SNP clustering at the time of map construction.

The remaining SNPs were used to generate individual parental maps for each family in JoinMap 4.1.^31^ SNPs were organized into linkage groups using a combination of independence tests using logarithm of odds scores from JoinMap output for individual families, consensus for these groupings between families and physical data for SNPs from the version 1.0 of the ‘Golden Delicious’ genome.^32^ SNPs that were observed to be in concordance with grouping with other SNPs based on logarithm of odds scores but were located on different chromosomes according to their physical position in the apple genome were kept in the groupings based on logarithm of odds scores.

Initial single parent genetic maps were created in JoinMap 4.1 through the software’s Monte Carlo maximum likelihood mapping algorithm approach.^31,33^ Linkage maps were oriented based on general SNP physical positions. SNPs were removed from these initial maps if they created gaps >50 centimorgans (cM). These initial single parent genetic maps were evaluated using graphical genotyping.^34^ This process is outlined in the Supplementary File #13 of Bassil *et al.* ^35^ Data were scanned for singletons, defined as double recombinant single points, that is, individual SNPs that displayed apparent recombination on both sides despite close linkage to adjacent markers. These singletons were recoded as missing values to achieve single-family SNP orders that minimized instances of probably spurious double recombination. Several SNPs that had many apparent singletons and poor-quality SNP score clustering were removed from analysis. The resolution of each individual parent’s map order was iteratively improved by increasing the levels of stringency until single parental maps were produced with fixed SNP orders containing no instances of double recombinations due to singletons or likely incorrect SNP order as evidenced through graphical genotyping.

An integrated SNP map for all SNPs from all families was built from the resulting 10 individual parental maps in four stages. In the first stage, a fixed marker order was created for all of the markers across all of the mapping families. Hereto initial marker orders were determined through the construction of a consensus map created using the LPmerge package^36^ in the statistical software R version 3.2.4.^37^ This initial marker order was refined iteratively, starting first with markers that were heterozygous in ‘Honeycrisp’. Following this, SNPs that were heterozygous in gradually fewer of the other parents were built into the order. Graphical genotyping was used at each step to order the SNPs in a way that approximated the consensus map created using LPmerge, but without instances of false double recombinations caused by incorrect map order. This ordering process was also aided by a draft of the linkage map created by Di Pierro *et al*.^26^ SNPs within areas of no recombination were ordered based on physical position information where possible from version 1.0 of the ‘Golden Delicious’ genome^32^ or SNP sequences blasted against version 3.0.a1 available on the Genome Database for Rosaceae (https://www.rosaceae.org/species/malus/malus_x_domestica/genome_v3.0.a1).^29^

For the second stage of the integrated map construction process, SNPs were organized into haplotype blocks (haploblocks). These haploblocks were designed to include sets of adjacent SNPs with no recombination among all mapping individuals. The physical positions of SNPs were used to help define the boundaries of haploblocks to ensure the resulting genetic position of any SNP within a region of no recombination was associated with the haploblock it was more closely linked to physically. Haploblock Aggregator (http://www.wageningenur.nl/en/show/HaploblockAggregator.htm) was then used in the third stage to aggregate the segregation information of individual SNPs into these haploblocks and to convert this haploblock-aggregated data from a ‘cross pollinated’ JoinMap formatted data set into a ‘back cross’ JoinMap formatted data set for simplified integration of all separate individual family maps, as described by Di Pierro *et al.* ^26^ In the final stage, the resulting data were incorporated back into JoinMap and Monte Carlo maximum likelihood mapping was again used along with fixed SNP orders for the haploblocks to calculate mapping distances in the integrated genetic map. Each individual marker within a haploblock was given that haploblock’s cM position.

Following the construction of this integrated genetic map, a highly curated SNP-genotyped and pedigree-connected apple data set comprising 540 individuals from the RosBREED project^25^ was used to help order the SNPs within blocks where no recombination was observed within the mapping families. The data set comprised a large, diverse and breeding-relevant panel of apple cultivars, selections, ancestors and unselected seedlings of various pedigree-connected families^38^ genotyped with the 8 K SNP array.^20^ Recombination events within this data set were evaluated using FlexQTL and Visual FlexQTL^39^ (www.flexqtl.nl). SNP order was altered if the reordering could resolve double recombination events observed in the pedigree-connected data set and not introduce double recombination events within the mapping set. Following this process, Haploblock Aggregator and JoinMap were again used to create a revised integrated map. Following publication of the Di Pierro *et al*.^26^ linkage map, 20 SNPs that were polymorphic in only one of the five families in this study that had insufficient physical position information available and were within large blocks of SNPs that were inherited together were reassigned to haploblocks to make this linkage map more consistent with the Di Pierro *et al*.^26^ linkage map. These changes were only made if they did not introduce additional double recombinations within the mapping populations and the pedigree-connected data set.

### Genetic analysis of the ‘Honeycrisp’ pedigree

The pedigree-connected genetic data set used in the map construction described above was also used to elucidate the pedigree of ‘Honeycrisp’ by offering a wide germplasm set that might hold the unknown parent of ‘Honeycrisp’ or earlier ancestors of this unknown parent, and by allowing the phasing of each individual’s SNPs through the comparison to directly related individuals. In the phasing process, the parameter MSegDelta was set 1, which instructed FlexQTL to not adjust parental calls in case of highly distorted segregation patterns.

SNP scores for individuals were changed in this data set if the change resolved the types of Mendelian-inconsistent SNP inheritance errors described above in map construction or if the change resolved cases of likely false double recombination, also known as Mendelian-consistent errors,^30^ that were probably due to incorrect SNP scores. This latter type of marker score error was observed when short (generally<1 cM) double recombinations, usually involving only single SNPs, were observed in phased SNP output from FlexQTL. An example of this type of error would be if an individual’s offspring all have *AB* scores for a single SNP and one parent’s SNP score is *AA* and the other is *AB* but closely linked SNPs do not show any evidence of segregation distortion. This type of error would not show as a typical inheritance error as the offspring could have inherited the *B* allele from the *AB* parent, but for each progeny to have a *B*-allele, half of the progenies would have a recombination on either side of this SNP allele (as long as closely linked SNPs did not have any type of segregation distortion in this region). In cases like this, the changing of the parental *AB* score into *BB* would resolve all issues. SNPs that had many inheritance errors or many cases of likely false double recombination were re-evaluated in GenomeStudio, which had become available at this stage, and SNP scores were revised or the SNP was removed from the data set. SNP scores that were suspected of being incorrect due to null alleles were recoded as missing data. Phased SNP data of this data set from FlexQTL output was used to validate the parenthood of ‘Keepsake’ and to further elucidate the ‘Honeycrisp’ pedigree.

A simple, manual unphased genotype-matching analysis compared SNP data of ‘Honeycrisp’, with its parent ‘Keepsake’ and likely candidate grandparents of ‘Honeycrisp’ using data from the initial noncurated pedigree-connected genetic data set. Likely candidate grandparents included individuals that were known to be used as breeding parents prior to the 1970s in the UMN apple breeding program and that were still available. In this analysis, unphased SNP data for a subset of SNPs deemed to have good clustering quality in GenomeStudio and organized by physical position were compared between ‘Honeycrisp’, its validated parent ‘Keepsake’ and the candidate grandparents to see whether SNP scores in ‘Honeycrisp’ could have arisen from ‘Keepsake’ and each candidate grandparent pair through manually conducting possible phasing of markers. Two likely grandparent candidates were identified. They were confirmed to be grandparents in further analyses using SNP data from the curated pedigree-connected genetic data set. Phased SNP data from these candidate grandparents and the parents of ‘Keepsake’, ‘Frostbite’ and ‘Northern Spy’ were evaluated to determine whether the SNP data from all of them could constitute the entirety of the phased SNP data from ‘Honeycrisp’ across a 3435 SNP subset of the integrated map.

Once these candidate grandparents were confirmed, the pedigree-connected genetic data set was searched for UMN selections that had these newly identified ‘Honeycrisp’ grandparents as recorded parents either in breeding program records or by analysis of the SNP data. These UMN selections were tested as being the unknown parent of ‘Honeycrisp’. Individuals were excluded *a priori* as parents if Mendelian-inconsistent and Mendelian-consistent errors composed >1% (35 SNPs) of their curated SNP data.

SNP data were evaluated from several UMN selections to determine if they share the unknown parent of ‘Honeycrisp’. This process was conducted in several steps. First, existing pedigree records for all UMN selections genotyped on the 8 K SNP array were evaluated for identity by descent consistency across the pedigree-connected data set by evaluating inheritance errors between them and their recorded parent(s). If at least one parent of a UMN selection was not identified, the SNP data for that selection were queried against the SNP data for all founding UMN selections and cultivars that could be possible parents. A parent was assigned to a selection if the parent-offspring relationship was historically concordant and exhibited <1% of SNPs with Mendelian-inconsistent errors and if those Mendelian-inconsistent errors could be resolved through steps outlined in the description of the RosBREED data set curation. UMN selections having one parent confirmed using SNP data and one remaining unknown parent were evaluated to determine whether the newly identified grandparents of ‘Honeycrisp’ were also probable grandparents of these selections. A selection was deemed to also share the newly identified ‘Honeycrisp’ grandparents if phased SNP data from the selection indicated that the unknown parent was composed entirely of haplotypes from phased SNP data of the newly identified ‘Honeycrisp’ grandparents with <1% of marker calls being Mendelian-inconsistent errors.

The UMN selections that matched the criteria for sharing the newly identified grandparents of ‘Honeycrisp’ through an unknown parent were then evaluated to determine whether ‘Honeycrisp’ and these selections shared this common unknown parent. The unknown parent of ‘Honeycrisp’ was determined to be shared with a UMN selection if the UMN selection and ‘Honeycrisp’ had between them, at maximum, only one of the two haplotypes from each of the newly identified grandparents of ‘Honeycrisp’ at any given locus, with a SNP mismatch rate of <1%. This determination was made through the evaluation of the unknown parent portion of the phased SNP data for ‘Honeycrisp’ and the UMN selection under consideration and the phased SNP data for the newly identified ‘Honeycrisp’ grandparents.

The haplotype composition and SNP scores of the unknown parent of ‘Honeycrisp’ were then imputed using phased SNP scores from the newly identified ‘Honeycrisp’ grandparents, ‘Honeycrisp’ and the UMN selections identified to also be offspring of the unknown parent of ‘Honeycrisp’. SNP scores for the unknown parent were coded as missing data when there was insufficient haplotype information from the offspring of the unknown parent, when there was missing data in the parents of the unknown parent, and when >50% of the offspring of the unknown parent had markers scores that were discordant with the marker scores of the parents of the unknown parent.

Finally, the original UMN selection records were evaluated to determine the likely identity of this unknown parent. Pedigree records of the UMN selections determined to share the unknown parent of ‘Honeycrisp’ were first evaluated and a likely parent was identified. Additional offspring of this likely parent were then identified and one was found to have been genotyped but not included in previous steps because both of its parents were not present in the pedigree-connected data set. The SNP calls for this individual and the imputed SNP calls for the unknown parent were evaluated for a parent-offspring relationship to validate the identity of the individual and its imputed SNP calls.

## RESULTS

### Integrated genetic map

The integrated genetic map created in this study includes 3 632 SNPs from the apple 8 K Illumina Infinium SNP array v1^20^ and spans a total length of 1 172 cM across 17 linkage groups (Complete genetic map and meta data for each SNP can be found in Supplementary Table 1). In the 413 individuals used for map development and scored for the 3 632 SNPs, 231 individual SNP scores (0.0154% of the total data) that were either double recombinant singletons or inconsistent were recoded as missing data. The average distance between SNPs in the genetic map is 0.32 cM. The average size of haploblocks is 3.14 SNPs and the average distance between them is 1.03 cM. Approximately 13% (464) of the SNPs mapped to different linkage groups than what was assigned in the *Malus*×*domestica* version 1.0 genome sequence assembly.

### Candidate grandparents of ‘Honeycrisp’

The simple, manual unphased genotype-matching analysis of candidate grandparents of ‘Honeycrisp’ using the initial noncurated pedigree-connected data set indicated that ‘Duchess of Oldenburg’ and ‘Golden Delicious’ were the most likely candidates. This analysis suggested that SNP scores for ‘Duchess of Oldenburg’ and ‘Golden Delicious’ provided a complementary fit for the SNP haplotype of the unknown parent when compared alongside ‘Honeycrisp’ and its known parent ‘Keepsake’.

### Validation of grandparents of ‘Honeycrisp’

**Figure 1:**
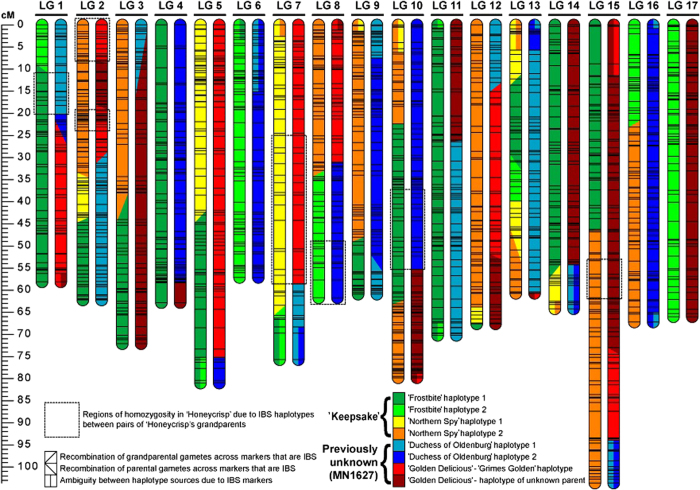
Haplotype composition and areas of recombination for parental and grandparental gametes for all 17 pairs of linkage groups of 'Honeycrisp' color coded for grandparental contribution from 'Frostbite' and 'Northern Spy' through parent ‘Keepsake’, and from 'Duchess of Oldenburg' and 'Golden Delicious' through the previously unidentified parent MN1627. Marker organization between linkage groups for grandparents that have no known parents is arbitrary as no parental data was available to organize them. Regions of uncertainty between haplotypes due to haplotypes that are identical by state (IBS) are shown via regions with two colors. Regions of homozygosity in 'Honeycrisp' that are due to haplotypes that are likely identical by descent from the grandparental pair 'Frostbite' and 'Duchess of Oldenburg' or the grandparental pair 'Northern Spy' and 'Golden Delicious' (see Supplementary Table 2) are highlighted by a dashed box around the region. These regions share at least 25 SNPs and 8 cM between each pair of grandparents. SNP, single-nucleotide polymorphism.

The identity by descent analysis of phased SNP data supported ‘Keepsake’ as a parent of ‘Honeycrisp’. The haplotype contribution from the parents of ‘Keepsake’ to the haplotype composition of ‘Honeycrisp’ is represented in Figure 1 and Supplementary Table 2 by the colors dark and light green for the possible haplotypes from ‘Frostbite’ and by yellow and orange for ‘Northern Spy’. This analysis also supported ‘Duchess of Oldenburg’ and ‘Golden Delicious’ as grandparents through the unknown parent of ‘Honeycrisp’. The haplotype contribution from these grandparents to the haplotype composition of ‘Honeycrisp’ is represented in Figure 1 and Supplementary Table 2 by the colors light and dark blue for the possible haplotypes from ‘Duchess of Oldenburg’ and by red for the haplotype from ‘Grimes Golden’ (recently reported as a parent of ‘Golden Delicious’)^40^ and dark red for the unknown parent of ‘Golden Delicious’. Extended SNP haplotypes for the two homologous chromosomes of each of the four grandparents were complementary such that they could compose the two homologous haplotypes of ‘Honeycrisp’ while showing logical meiosis evidence. This pedigree was free of Mendelian-consistent and Mendelian-inconsistent errors across all SNPs evaluated. The pedigree also included few missing data from ‘Duchess of Oldenburg’ (53 SNPs with missing data, 1.54% of the total SNP data), ‘Golden Delicious’ (6 SNPs, 0.17%), ‘Keepsake’ (56 SNPs, 1.63), ‘Northern Spy’ (58 SNPs, 1.69) and ‘Frostbite’ (35, 1.02%) (shown by dash symbols in Supplementary Table 2).

### Identification of siblings of ‘Honeycrisp’

Two extant UMN selections had ‘Duchess of Oldenburg’ and ‘Golden Delicious’ recorded as parents with SNP data supporting this parentage: MN1478 and ‘Red Baron’. However, both individuals were eliminated as possible parents of ‘Honeycrisp’ because of high numbers of Mendelian-inconsistent errors when tested as possible parents. Next, four UMN selections (MN1708, MN1789, MN1837 and MN1888) were observed to have one identified parent for which SNP data was available and one unknown parent that was consistent with itself being the offspring of a cross between ‘Duchess of Oldenburg’ and ‘Golden Delicious’. The phased SNP data strongly suggested that these four selections and ‘Honeycrisp’ indeed share a common parent (Supplementary Table 3). Mendelian-consistent errors were observed in only 61 cases across 38 SNPs (<1% of SNP scores per individual, depicted as yellow shaded areas in Supplementary Table 3) among the four selections and the new putative parentage. These SNPs were re-evaluated in GenomeStudio to identify causes of the discrepancies. Four errors were due to missing parental SNP data that were incorrectly imputed by FlexQTL. Five errors were due to incorrect parental marker scores (one each for ‘Golden Delicious’, ‘Keepsake’ and ‘Duchess of Oldenburg’ and two for MN1691). The remaining 29 errors were due to poor SNP clustering, of which 12 were likely due to the presence of additional clusters other than *AA*, *AB* and *BB*, which confounded the SNP scores, and 3 were likely due to the presence of null alleles. These types and numbers of Mendelian-consistent errors were similar to those for other individuals in the pedigree-connected data set that had validated parent-child relationships. MN1708, MN1789, MN1837 and MN1888 were thus confirmed as siblings of ‘Honeycrisp’ via its unknown parent.

### Identification of unknown parent of ‘Honeycrisp’

MN1789 was recorded as an offspring from MN1736×‘Beacon’, but was determined to be an offspring from MN1691×(‘Duchess of Oldenburg’×‘Golden Delicious’). MN1708, like ‘Honeycrisp’, was originally recorded as ‘Honeygold’×‘Macoun’ but SSR markers examined by Cabe *et al.* ^16^ suggested MN1708 was derived from ‘Keepsake’×unknown parent, which was confirmed in this study. MN1837 and MN1888 were recorded as offspring from MN1691 (‘Goodland’×‘Fireside’)×MN1627. MN1691 was confirmed to be a parent of MN1837 and MN1888. MN1627 was recorded as an offspring from ‘Duchess of Oldenburg’ (as the sport ‘Daniel’s Red Duchess’)×‘Golden Delicious’ and was selected in 1951. Unfortunately, trees of this selection no longer exist in the UMN apple breeding program. The last recorded distribution of MN1627 was in 1965. We identified current contacts for recipients, where possible, and queried them about the presence of MN1627. All responding recipients indicated it was no longer present in their orchards or collections.

**Figure 2:**
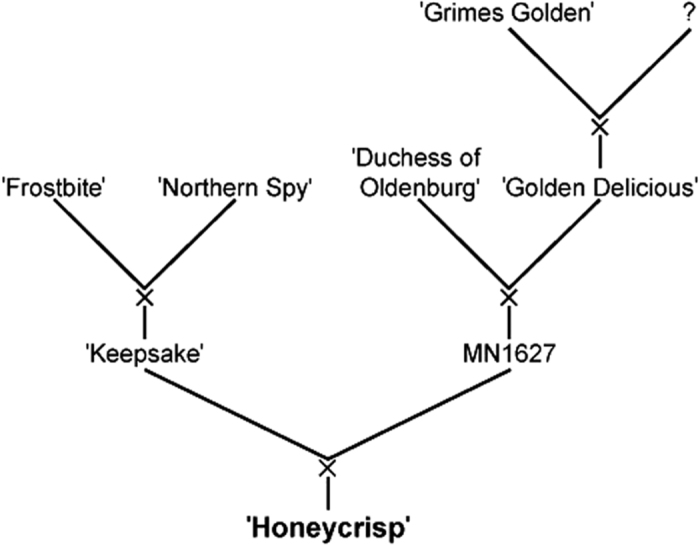
Reconstructed pedigree for 'Honeycrisp' supported by this study. Maternal DNA was not evaluated in this study, meaning the order of parents and grandparents from left to right within crosses is arbitrary.

The confirmed breeding records for MN1837 and MN1888, coupled with the finding that these individuals are half-sibs of ‘Honeycrisp’ (Supplementary Table 3) suggests that the previously unknown parent of ‘Honeycrisp’ is the probably extinct UMN apple selection MN1627, resulting in the reconstructed pedigree shown in Figure 2.

### Genetic characterization of MN1627 through haplotype and SNP imputation

The availability of SNP data for five siblings and both identified parents of MN1627 allowed for the imputation of 97.8% of its SNP scores across the 3 435 SNPs under consideration (columns D and G of Supplementary Table 3). There were about 100 cM of haplotypes across six linkage groups (8.5% of the genome) that were unable to be imputed due to lack of representation of either ‘Duchess of Oldenburg’ or ‘Golden Delicious’ from offspring of MN1627. However, SNPs that were homozygous in regions that did not have haplotype representation were still able to be imputed, which is what accounted for the discrepancy between the higher percentage of the SNPs imputed verses the lower percent coverage of the imputed haplotypes.

Imputed SNP scores of MN1627 were validated by examining the genotype of MN1839, which was recorded as an offspring from MN1627×‘Prima’. SNP data for ‘Prima’ provided from the FruitBreedomics project^41^ was observed to not match as a parent of MN1839 despite this sample of ‘Prima’ having proven to be true to type.^42^ However, there were no Mendelian-inconsistent errors in a parent-offspring relationship evaluation between MN1839 and the imputed SNP data for MN1627, thus providing further confirming evidence that MN1839 is a half-sibling of ‘Honeycrisp’ and validating the imputed genotype of MN1627.

### Homozygous genomic regions of ‘Honeycrisp’

‘Honeycrisp’ was detected to have several large regions of homozygosity. The identity by descent analysis using phased SNP data revealed that most of this homozygosity is attributed to shared haplotypes between ‘Frostbite’ and ‘Duchess of Oldenburg’ and between ‘Northern Spy’ and ‘Golden Delicious’ (represented by shaded areas in Supplementary Table 2). The extended SNP haplotypes that are identical by state for >25 SNPs and 8 cM between ‘Golden Delicious’ and ‘Northern Spy’ span ~21% of the phased marker data between ‘Northern Spy’ and ‘Golden Delicious’ and include regions up to 160 SNPs and 38 cM (represented by areas shaded by diagonal lines in ‘Northern Spy’ and ‘Golden Delicious’ columns in Supplementary Table 2). These extended SNP haplotypes can be found in either the phased SNP data from both ‘Grimes Golden’^40^ or the unknown parent of ‘Golden Delicious’. ‘Frostbite’ has extended SNP haplotypes that are identical by state with ‘Duchess of Oldenburg’ for ~29% of the genome. These identical by state SNP haplotypes span the entirety of one homolog of each of linkage groups 12 and 16 and large portions of other linkage groups (represented by areas shaded by small dots in ‘Frostbite’ and ‘Duchess of Oldenburg’ columns in Supplementary Table 2).

## DISCUSSION

### The pedigree of ‘Honeycrisp’

The pedigree of ‘Honeycrisp’ (Figure 2) was deduced to be ‘Keepsake’×MN1627 (‘Duchess of Oldenburg’×‘Golden Delicious’) (haplotype composition represented in graphically in Figure 1 and through phased marker data shown in Supplementary Table 2) through the use of a high-quality integrated genetic map and a large pedigree-connected data set. This study, based on 3435 SNPs, confirmed a previous report supporting ‘Keepsake’ as one parent of ‘Honeycrisp’ ^16^ that was based on 11 SSR markers. The identification of ‘Duchess of Oldenburg’ and ‘Golden Delicious’ as grandparents is significant because they are of worldwide importance and this finding connects the pedigree of ‘Honeycrisp’ to the pedigrees of many internationally important cultivars that descend from them. ‘Duchess of Oldenburg’, also known as ‘Borowitsky’, ‘Borovitsky’ and ‘Charlamowski’, was introduced into the United States from England in 1835, where it had been earlier brought from Russia in the early 1800s.^43^ ‘Duchess of Oldenburg’ was used extensively for breeding in the formative years of the UMN apple breeding program^44^ because of its extreme winter hardiness. ‘Golden Delicious’ originated around 1890 in West Virginia and was released commercially in 1916.^45^ ‘Golden Delicious’ is an ancestor of a multitude of important cultivars^46^ including ‘Gala’ (‘Kidd’s Orange Red’×‘Golden Delicious’), ‘Jonagold’ (‘Golden Delicious’×‘Jonathan’) and ‘Cripps Pink’ (‘Golden Delicious’×‘Lady Williams’)^19^ and is famous in the scientific community for being the first sequenced apple genome.^32^

The previously unknown parent of ‘Honeycrisp’, MN1627, was indirectly identified through the analysis of phased SNP data of five UMN selections identified to be siblings of ‘Honeycrisp’, three of which had MN1627 listed as parents in the original selection records. MN1627 was recorded as being a cross between ‘Duchess of Oldenburg’ and ‘Golden Delicious’. Historical breeding records indicated that crosses between ‘Duchess of Oldenburg’ and ‘Golden Delicious’ were made numerous times in the UMN apple breeding program from the 1920s through the 1930s and had resulted in several selections and the cultivar Red Baron,^47^ released in 1970. Many of these selections would have been available for breeding at the time of the cross that begat ‘Honeycrisp’.

### Possible inbreeding within ‘Honeycrisp’ and genetic relatedness among ancestors

This study also revealed that many large regions of homozygosity in the ‘Honeycrisp’ genome (represented by dashed rectangles in Figure 1) are due to likely close genetic relationships between ‘Frostbite’ and ‘Duchess of Oldenburg’ and between ‘Northern Spy’ and ‘Golden Delicious’ (represented by shaded areas in Supplementary Table 2), indicating shared ancestry between the parents of ‘Honeycrisp’ and leading to some degree of inbreeding for ‘Honeycrisp’. The shared extended haplotypes between ‘Northern Spy’ and ‘Golden Delicious’ contain haplotypes from both the known parent, ‘Grimes Golden’,^40^ and the unknown parent of ‘Golden Delicious’. ‘Northern Spy’ originated in New York in the early 1800s,^45^ ‘Grimes Golden’ originated in West Virginia in the 1790s, and ‘Golden Delicious’ originated in West Virginia in the 1890s,^45^ making it chronologically and geographically possible that ‘Northern Spy’, ‘Grimes Golden’ and the unknown parent of ‘Golden Delicious’ share one or more recent common ancestors. The relatively large extended regions of shared SNP haplotypes between ‘Frostbite’ and ‘Duchess of Oldenburg’ suggest that ‘Frostbite’ is a grandchild of ‘Duchess of Oldenburg’. ‘Frostbite’ was released in 2008 but was originally selected in 1922.^48^ It is thought that the tree originated from open-pollinated seeds of ‘Malinda’ that were described by Dorsey,^49^ however ‘Malinda’ was determined to not be the parent of ‘Frostbite’.^16^ The time of the origin of ‘Frostbite’ coincides with the presence and use of ‘Duchess of Oldenburg’ in the UMN breeding program.^44,49^ That timing, in combination with the proportion of haplotype sharing, indicates it is a likely possibility that ‘Frostbite’ is a grandchild of ‘Duchess of Oldenburg’.

### Integrated linkage map quality

The quality of the integrated linkage map used in this study (found in Supplementary Table 1) was vital to the findings as high numbers of false marker orders would have prevented accurate marker phasing across the pedigree-connected data set, which would have impeded identification of pedigree relationships discovered and detailed in this study. The high quality of the current linkage map should make it useful to future studies that use data from the apple 8, 20 and 480 K SNP arrays.^20–22^ Both this map and the 20 K iGL map^26^ are useful because each has a unique SNP composition. Indeed, of the currently mapped 3632 SNPs, 1441 are unique to the current map and 2191 were in common with the 20 K iGL map.

The quality of the map was achieved because of intense data curation and the approach in map construction, which was similar to that proposed by Di Pierro *et al*.^26^ The methods used to construct these linkage maps differ from previous apple linkage maps made with data from apple SNP arrays^28,50,51^ by using graphical genotyping^34^ to avoid double recombinations along with the use of multiple families and the newly developed tool Haploblock Aggregator (http://www.wageningenur.nl/en/show/HaploblockAggregator.htm) to create an integrated genetic map that reduced cases of false marker order. The high quality of our current map and the 20 K iGL map is underlined by the low number of SNPs that are in discordant order (71 SNPs, 3.2%), the small size of the genetic segments in which these discordant orders occurred (usually <0.5 cM, data not shown), and the similar small size of both genetic maps. The map created in this study is 76 cM smaller than that of Di Pierro *et al.*,^26^ which is likely a function of a lower representation of the chromosomal ends and the smaller numbers of families used here. The similarity of these maps in size and map order will be useful for the transferability of genetic data across studies using apple SNP array data.

## CONCLUSION

This study provides a revised pedigree for ‘Honeycrisp’ that is consistent across a pedigree-connected data set using a new dense and high-quality integrated SNP map. This pedigree and the identification of relatedness between two pairs of ‘Honeycrisp’s grandparents will be useful in future genetic studies involving ‘Honeycrisp’. The haplotype and SNP data for the newly identified parent of ‘Honeycrisp’, MN1627, has been imputed and made available in this study (Supplementary Table 3). Though no longer available, the imputed haplotype and SNP data for MN1627 will enable accurate tracing of the grandparental allelic contributions inherited by ‘Honeycrisp’ at any given region of its genome. Identifying and using these types of relationships for pedigree-based quantitative trait locus analyses is an explicit approach of RosBREED and these results will be useful in this work. The discovery that ‘Duchess of Oldenburg’ and ‘Golden Delicious’ are grandparents of ‘Honeycrisp’ connects the pedigree of ‘Honeycrisp’ to many cultivars of worldwide significance. The ability to connect these pedigrees will result in more accurate results from pedigree-based quantitative trait locus analyses to understand the genetic underpinning of ’Honeycrisp’s traits, such as its highly acclaimed crisp texture, its reported susceptibility to developing leaf chlorosis and soft scald and its reported apple scab resistance. The findings described in this paper are expected to help in the development of future superior apple cultivars related to ‘Honeycrisp’.

## References

[bib1] Gallardo RK , Hanrahan I , Hong YA , Luby JJ . Crop load management and the market profitability of ‘Honeycrisp’ apples. Hort Technol 2015; 25: 575–584.

[bib2] Luby J , Bedford DS . Apple tree: Honeycrisp. Regents of the University of Minnesota, assignee, US patent, US PP7197, 1990.

[bib3] Mann H , Bedford D , Luby J , Vickers Z , Tong C . Relationship of instrumental and sensory texture measurements of fresh and stored apples to cell number and size. Hortscience 2005; 40: 1815–1820.

[bib4] Tong C , Krueger D , Vickers Z , Bedford D , Luby J , El-Shiekh A et al. Comparison of softening-related changes during storage of ‘Honeycrisp' apple, its parents, and ‘Delicious'. J Am Soc Hort Sci 1999; 124: 407–415.

[bib5] Rosenberger D , Schupp J , Watkins C , Iungerman K , Hoying S , Straub D et al. Honeycrisp: promising profit maker or just another problem child. NY Fruit Quarterly 2001; 9: 9–13.

[bib6] Trujillo DI , Mann HS , Tong CB . Examination of expansin genes as related to apple fruit crispness. Tree Genet Genomes 2012; 8: 27–38.

[bib7] Clark MD , Bus VG , Luby JJ , Bradeen JM . Characterization of the defence response to *Venturia inaequalis* in ‘Honeycrisp’ apple, its ancestors, and progeny. Eur J Plant Pathol 2014; 140: 69–81.

[bib8] Bedford DS , Luby J . Apple tree named ‘Minneiska’. Regents of the University of Minnesota, assignee. US patent, US PP18812, 2008.

[bib9] Brown SK , Maloney K. Apple tree named ‘New York 1’. US patent, US PP22228, 2011.

[bib10] Nystrom C . Apple tree, ‘CN B60’. US patent, US PP23862, 2013.

[bib11] Nystrom C . Apple tree ‘CN 121’. US patent, US PP23777, 2013.

[bib12] Shefelbine D . Apple tree ‘DS 22’. US patent, US PP23933, 2013.

[bib13] Evans KM , Barritt BH , Konishi BS , Brutcher LJ , Ross CF . ‘WA 38’ apple. Hortscience 2012; 47: 1177–1179.

[bib14] Dodd W , Doud D , Lynd JM , Miller G . Apple tree named ‘MAIA1’. Midwest Apple Improvement Association, assignee. US patent, US PP24579, 2014.

[bib15] Bedford D , Luby J . Apple tree named ‘MN55’. Regents of the University of Minnesota, assignee. US patent, US PP26412, 2016.

[bib16] Cabe PR , Baumgarten A , Onan K , Luby JJ , Bedford DS . Using Microsatellite Analysis to Verify Breeding Records: A study of ‘Honeycrisp' and Other Cold-hardy Apple Cultivars. Hortscience 2005; 40: 15–17.

[bib17] Savolainen V , Corbaz R , Moncousin C , Spichiger R , Manen JF . Chloroplast DNA variation and parentage analysis in 55 apples. Theor Appl Genet 1995; 90: 1138–1141. 24173075 10.1007/BF00222934

[bib18] Harada T , Matsukawa K , Sato T , Ishikawa R , Niizeki M , Saito K . DNA-RAPDs detect genetic variation and paternity in Malus. Euphytica 1992; 65: 87–91.

[bib19] Evans KM , Patocchi A , Rezzonico F , Mathis F , Durel CE , Fernández-Fernández F et al. Genotyping of pedigreed apple breeding material with a genome-covering set of SSRs: trueness-to-type of cultivars and their parentages. Mol Breed 2011; 28: 535–547.

[bib20] Chagné D , Crowhurst RN , Troggio M , Davey MW , Gilmore B , Lawley C et al. Genome-wide SNP detection, validation, and development of an 8 K SNP array for apple. PLoS ONE 2012; 7: e31745. 22363718 10.1371/journal.pone.0031745 PMC3283661

[bib21] Bianco L , Cestaro A , Sargent DJ , Banchi E , Derdak S , Di Guardo M et al. Development and validation of a 20 K single nucleotide polymorphism (SNP) whole genome genotyping array for apple (*Malus×domestica* Borkh). PLoS ONE 2014; 9: e110377. 25303088 10.1371/journal.pone.0110377 PMC4193858

[bib22] Bianco L , Cestaro A , Linsmith G , Muranty H , Denancé C , Théron A et al. Development and validation of the Axiom Apple 480 K SNP genotyping array. Plant J 2016; 86: 62–74. 26919684 10.1111/tpj.13145

[bib23] Pikunova A , Madduri M , Sedov E , Noordijk Y , Peil A , Troggio M et al. ‘Schmidt’s Antonovka’ is identical to ‘Common Antonovka’, an apple cultivar widely used in Russia in breeding for biotic and abiotic stresses. Tree Genet Genomes 2013; 10: 261–271.

[bib24] Rosyara UR , Sebolt AM , Peace C , Iezzoni AF . Identification of the Paternal Parent of ‘Bing’ Sweet Cherry and Confirmation of Descendants Using Single Nucleotide Polymorphism Markers. J Am Soc Hortic Sci 2014; 139: 148–156.

[bib25] Iezzoni AC , Weebadde C , Luby J , Yue C , van de Weg E , Fazio G et al. RosBREED: enabling marker-assisted breeding in Rosaceae. Acta Hortic 2010; 859: 389–394.

[bib26] Di Pierro AE , Gianfranceschi L , Di Guardo M , Koehorst-van Putten HJJ , Kruisselbrink JW , Longhi S et al. A high-density, multi-parental SNP genetic map on apple validates a new mapping approach for outcrossing species. Hortic Res 2016; 3: 16057. 27917289 10.1038/hortres.2016.57 PMC5120355

[bib27] McKay SJ , Bradeen JM , Luby JJ . Prediction of genotypic values for apple fruit texture traits in a breeding population derived from ‘Honeycrisp’. J Am Soc Hortic Sci 2011; 136: 408–414.

[bib28] Clark MD , Schmitz CA , Rosyara UR , Luby JJ , Bradeen JM . A consensus ‘Honeycrisp’ apple (*Malus×domestica*) genetic linkage map from three full-sib progeny populations. Tree Genet Genomes 2014; 10: 627–639.

[bib29] Jung S , Ficklin SP , Lee T , Cheng CH , Blenda A , Zheng P et al. The Genome Database for Rosaceae (GDR): year 10 update. Nucleic Acids Res 2014; 42 (D1): D1237–D1244. 24225320 10.1093/nar/gkt1012 PMC3965003

[bib30] Sobel E , Papp JC , Lange K . Detection and integration of genotyping errors in statistical genetics. Am J Hum Genet 2002; 70: 496–508. 11791215 10.1086/338920 PMC384922

[bib31] Van Ooijen JW . JoinMap 4, Software for the calculation of genetic linkage maps in experimental populations. Kyazma BV, Wageningen, 2006; 33: 10–371.

[bib32] Velasco R , Zharkikh A , Affourtit J , Dhingra A , Cestaro A , Kalyanaraman A et al. The genome of the domesticated apple (*Malus x domestica* Borkh.). Nat Genet 2010; 42: 833–839. 20802477 10.1038/ng.654

[bib33] Jansen J , de Jong AG , van Ooijen JW . Constructing dense genetic linkage maps. Theor Appl Genet 2001; 102: 1113–1122.

[bib34] Young ND , Tanksley SD . Restriction fragment length polymorphism maps and the concept of graphical genotypes. Theor Appl Genet 1989; 77: 95–101. 24232480 10.1007/BF00292322

[bib35] Bassil NV , Davis TM , Zhang H , Ficklin S , Mittmann M , Webster T et al. Development and preliminary evaluation of a 90 K Axiom SNP array for the allo-octoploid cultivated strawberry Fragaria×ananassa. BMC Genomics 2015; 16: 1. 25886969 10.1186/s12864-015-1310-1 PMC4374422

[bib36] Endelman JB , Plomion C . LPmerge: an R package for merging genetic maps by linear programming. Bioinformatics 2014; 30: 1623–1624. 24532720 10.1093/bioinformatics/btu091

[bib37] R Core Team. R: A language and environment for statistical computing. R Foundation for Statistical Computing: Vienna, Austria. 2016. Available at https://www.R-project.org/.

[bib38] Peace CP , Luby JJ , van de Weg WE , Bink MC , Iezzoni AF . A strategy for developing representative germplasm sets for systematic QTL validation, demonstrated for apple, peach, and sweet cherry. Tree Genet Genomes 2014; 10: 1679–1694.

[bib39] Bink MCAM , Jansen J , Madduri M , Voorrips RE , Durel C-E , Kouassi AB et al. Bayesian QTL analyses using pedigreed families of an outcrossing species, with application to fruit firmness in apple. Theor Appl Genet 2014; 127: 1073–1090. 24567047 10.1007/s00122-014-2281-3

[bib40] Salvi S , Micheletti D , Magnago P , Fontanari M , Viola R , Pindo M et al. One-step reconstruction of multi-generation pedigree networks in apple (*Malus×domestica* Borkh.) and the parentage of Golden Delicious. Mol Breed 2014; 34: 511–524.

[bib41] Laurens F , Durel CE , Patocchi A , Peil A , Salvi S , Tartarini S et al. Review on apple genetics and breeding programmes and presentation of a new European initiative to increase fruit breeding efficiency. J Fruit Sci 2010; 27: 102–107.

[bib42] Voorrips RE , Bink MCAM , Kruisselbrink JW , Koehorst- van Putten HJJ , van de Weg WE . PediHaplotyper: Software for consistent assignment of marker haplotypes in pedigrees. Mol Breed 2016; 36: 119. 27547106 10.1007/s11032-016-0539-y PMC4977329

[bib43] Beach SA , Booth NO , Taylor OM . Apples of New York (Vol II) State of New York—Department of Agriculture. J. B. Lyon Company: New York, 1905, pp 150–152.

[bib44] Dorsey MJ . The set of fruit in apple crosses. Proc Amer Soc Hortic Sci 1921; 18: 82–94.

[bib45] Morgan J , Richards A . The Book of Apples. Ebury Press: London, UK. 1993.

[bib46] Noiton DAM , Alspach PA . Founding clones, inbreeding, coancestry and status number of modern apple cultivars. J Amer Soc Hortic Sci 1996; 121: 773–782.

[bib47] Stushnoff C , Munson ST , Hertz LB , Pellett HM . Honeygold and Red Baron, two new hardy apples from Minnesota. Fruit Var Hortic Dig 1969; 23: 63–64.

[bib48] Clark JR , Finn CE . Register of new fruit and nut cultivars list 45. Hortscience 2010; 45: 716–756.

[bib49] Dorsey MJ . Some characteristics of open-pollinated seedlings of the Malinda apple. Proc Amer Soc Hortic Sc 1919; 16: 36–42.

[bib50] Antanaviciute L , Fernández-Fernández F , Jansen J , Banchi E , Evans KM , Viola R et al. Development of a dense SNP-based linkage map of an apple rootstock progeny using the Malus Infinium whole genome genotyping array. BMC Genomics 2012; 13: 203. 22631220 10.1186/1471-2164-13-203 PMC3410780

[bib51] Troggio M , Šurbanovski N , Bianco L , Moretto M , Giongo L , Banchi E et al. Evaluation of SNP data from the Malus infinium array identifies challenges for genetic analysis of complex genomes of polyploid origin. PloS ONE 2013; 8: e67407. 23826289 10.1371/journal.pone.0067407 PMC3694884

